# Stem Cells for Bone Regeneration: From Cell-Based Therapies to Decellularised Engineered Extracellular Matrices

**DOI:** 10.1155/2016/9352598

**Published:** 2016-02-21

**Authors:** James N. Fisher, Giuseppe M. Peretti, Celeste Scotti

**Affiliations:** ^1^IRCCS Istituto Ortopedico Galeazzi, 20161 Milan, Italy; ^2^Dipartimento di Scienze Biomediche per la Salute, Università degli Studi di Milano, 20122 Milan, Italy

## Abstract

Currently, autologous bone grafting represents the clinical gold standard in orthopaedic surgery. In certain cases, however, alternative techniques are required. The clinical utility of stem and stromal cells has been demonstrated for the repair and regeneration of craniomaxillofacial and long bone defects although clinical adoption of bone tissue engineering protocols has been very limited. Initial tissue engineering studies focused on the bone marrow as a source of cells for bone regeneration, and while a number of promising results continue to emerge, limitations to this technique have prompted the exploration of alternative cell sources, including adipose and muscle tissue. In this review paper we discuss the advantages and disadvantages of cell sources with a focus on adipose tissue and the bone marrow. Additionally, we highlight the relatively recent paradigm of developmental engineering, which promotes the recapitulation of naturally occurring developmental processes to allow the implant to optimally respond to endogenous cues. Finally we examine efforts to apply lessons from studies into different cell sources and developmental approaches to stimulate bone growth by use of decellularised hypertrophic cartilage templates.

## 1. Introduction

Bone tissue is capable of spontaneous self-repair, with no scarring, generating new tissue that is all but indistinguishable from surrounding bone. However, in certain circumstances, the defect is too large (due to tumour resection, osteomyelitis, atrophic nonunions, and periprosthetic bone loss), or the underlying physiological state of the patient impairs natural healing (osteoporosis, infection, diabetes, and smoking) necessitating intervention. Autologous bone grafting is today the gold standard for bone repair, although the costs of this approach are considerable due to the additional surgical procedures required to harvest the bone material, the consequent donor site morbidity [1], and the risk of infection and complications. Additionally, this approach is hampered by the limited amount of donor material available for transplantation which can be prohibitive when dealing with large defects. To resolve these issues, both allograft- and xenograft-based strategies have been proposed; however the risk of rejection in the former and of zoonoses in the latter has reduced their clinical impact. Bone tissue engineering (BTE) is an alternative strategy that has been explored to fill the clinical need for autologous bone transplantation.

Almost half a century has passed since the demonstration that ectopic transplantation of bone marrow and bone fragments leads to the formation of* de novo *bone tissue which, when transplanted subcutaneously, is later filled with bone marrow [2, 3]. Nowadays, the notion that a set of cells present in the bone marrow stroma can be cultured* in vitro* and can regenerate fully functional bone organs* in vivo* is well accepted, although the identity and precise molecular characterisation of the cell population responsible are still a matter of study and debate (reviewed in [4, 5]). The* ex vivo* expansion and manipulation of stromal cells derived from various sources form the foundation of the majority of current bone tissue engineering attempts to meet the clinical demands for bone regeneration and repair.

Over the last 50 years, the BTE field has made significant advances towards overcoming the limitations of conventional methods which is particularly relevant when an underlying pathology calls for alternatives to the* status quo*. Clinically, several examples of successful application of tissue engineering techniques to bone reconstruction exist within the literature [6–8]; however, on the whole, advances in basic science have not translated well into significantly increased clinical application. The reasons are, in part, financial, but additional problems such as low efficiency of differentiation, intrapatient variability [9], the risk of ectopic bone growth [10], possible transformation [11], or epithelial to mesenchymal transition coupled with an incomplete understanding of the underlying pathways which are being manipulated with factors, such as transforming growth factor *β* (TGF-*β*) and bone morphogenic proteins (BMPs) [10, 12–15], certainly play a role.

Minimal clinical adoption has prompted the exploration and adaptation of alternative methods including the use of stromal cells from nonbone sources [16, 17], most commonly, adipose tissue [8, 18–20], but also muscle [17]; the development of new tissue engineering paradigms in which the focus is shifted from “cells + cytokines” to the engineering and* in vitro* optimisation of treatments as a means to support* in vivo *developmental processes by harnessing innate developmental pathways [21–26]; and finally, attempts to create “off-the-shelf” products to stimulate the regeneration of bone through adoption of developmental engineering principles [27–29]. The various merits of these points will be the focus of this review.

## 2. Cell Source

While the bone marrow (BM) represents the most well-documented source of cells for the regeneration and repair of skeletal tissues, a wide variety of alternatives, including adipose tissue (AT) [18, 19], muscle [17], umbilical cord blood [16, 30], umbilical cord Wharton's jelly [31], dental pulp [32], and periosteal tissue [33], have been explored for bone regeneration. For the purpose of this review, we will focus on two sources of stromal cells which have been the subject of the greatest number of studies in recent years and which are both attractive for different reasons, namely, the bone marrow and adipose tissue.

### 2.1. From Bone Fragment Transplants to Identification of the Skeletal Stem Cell

In the late 1960s it was shown that bone fragments and/or suspensions of cultured bone marrow cells, when ectopically implanted in mice, rats, rabbits, and guinea-pigs, were capable of forming bone composed of donor osteoblasts, osteocytes, and bone marrow stroma adipocytes, which was capable of attracting host haematopoietic cells to the bone marrow stroma [3, 34]. It was later shown that, by plating cultured, nonhaematopoietic, bone marrow suspensions at low density, a specific subpopulation of plastic-adherent fibroblast-like cells could be isolated that were responsible for single-cell colony formation, the colony-forming unit-fibroblast (CFU-f) [35, 36]. It was clear that a nonhaematopoietic cell population within the bone marrow was responsible for the* in vivo* regeneration and spatial organisation of skeletal tissues.

In the early 1990s Arnold Caplan's group showed that rat bone marrow-derived mesenchymal cells, purified through plastic adherence, could be passaged multiple times, demonstrating self-renewal (albeit* in vitro*), and were still capable of differentiation into cells of the skeletal system* in vivo*, namely, osteoblasts and chondrocytes, and coined the term “mesenchymal stem cell” [37, 38]. Further studies in humans confirmed the ability of a rapidly dividing subset of bone marrow-derived stromal cells (BMSCs) to differentiate into skeletal lineages (bone, cartilage, adipocytes, and marrow stroma) [39, 40] in a hierarchical manner and to undergo* in vitro* self-renewal, giving rise to secondary colonies upon replating at the clonal level [41, 42].

*In vivo* demonstration of BMSC stem cell characteristics, namely, self-replication and multipotency, came with the description of CD146+/MCAM (melanoma cell adhesion molecule) [43] and nestin^+^ [44] perivascular adventitial cells. Transplantation of single CFU-f-derived CD146^+^ colonies implanted in hydroxyapatite-tricalcium phosphate (HA-TCP) carrier in a fibrin gel in mice resulted in the formation of ossicles with a functional bone marrow populated by murine (host) haematopoietic cells and endothelium with human CD146^+^ adventitial cells lining the sinusoidal vessels, which were capable of generating secondary CFU-fs* in vitro* [43]. Similarly, implantation of nestin^+^ clonal cell spheres harvested two months after subcutaneous implantation in mice resulted in the generation of secondary ossicles with donor-derived osteoblasts and nestin^+^ cells after eight months [44]. Nestin^+^ cells were shown to spatially associate with haematopoietic stem cells (HSCs), to express high levels of HSC maintenance genes, and to influence HSC homing in addition to differentiation into osteochondral lineages; in addition they were shown to be entirely responsible for the clonogenic activity of the CD45^−^ cell fraction [44]. More recently, evidence for a skeletal stem cell (SSC) resident in the BM reticulum, characterised by expression of the BMP antagonist Gremlin-1, has emerged [45] which has challenged previous ideas about the identity of the SSC, particularly the use of nestin as an appropriate SSC marker and the developmental origins of BM adipocytes [45], although it is possible that these conflicting data may be due to different active populations of SSCs during different phases of development [45, 46].

### 2.2. Clinical BTE Application of BMSCs

Practically, BMSCs are applicable to large bone defects in both small [47] and large [48, 49] animals when implanted within hydroxyapatite-based scaffolds. Experimental evidence for the ability of BMSCs to repair bone defects was given crucial clinical support in 2001, when Quarto and colleagues published results obtained in three patients with various long bone defects [6]. BMSCs were isolated and expanded* ex vivo* under the stimulation of specific growth factors [50] before implantation on hydroxyapatite (HA) scaffolds tailored to the dimensions of each bone defect. It was reported that “all patients recovered limb function” and that, within two months of implantation, good integration with the recipient bone was observed [6]. Shortly afterwards, the use of autologous BM encased within a titanium cage with bone mineral blocks for reconstructive mandibular reconstruction was reported [51]. The scaffold was implanted in the dorsal* latissimus dorsi* muscle for seven weeks allowing for growth and vascularisation before transplantation of the bone-muscle flap. This method, while slow, avoided the creation of a donor site bone defect. More recently, the successful treatment of long bone defects in four patients was reported after 6-7 years of follow-up [52]. As of the time of writing, 33 clinical trials (https://www.clinicaltrials.gov/) are registered for the use of BMSCs, only two of which are directed towards bone repair or regeneration: NCT02177565 is investigating the use of* in vitro *expanded autologous BMSCs for the treatment of nonunions although at the time of writing the trial has been completed, but no results are posted. The multicentre ORTHO-2 trial for the “Evaluation of Mesenchymal Stem Cells to Treat Avascular Necrosis of the Hip” (NCT02065167), as part of the REBORNE (regenerating bone defects using new biomedical engineering approaches) programme, for the use of autologous BMSCs for the treatment of necrosis of the femoral head got underway in late 2014; however no results are available as of yet. The paucity of clinical trials investigating the potential of autologous BMSCs for bone repair and regeneration likely reflect hurdles to clinical use, be it GMP cell expansion, interpatient variability, or the difficulty in enrolling sufficient patients, notwithstanding positive results previously reported [6].

### 2.3. Bone from Fat: Successful Application of Fat-Derived Cells for BTE

Concurrent with studies illustrating the clinical application of BMSCs for bone regeneration, it was demonstrated that human processed lipoaspirate (PLA) cells, isolated from liposuction procedures, could be induced to differentiate into osteogenic, adipogenic, chondrogenic, and myogenic lineages through incubation in specific media [18] and showed increased expression of core-binding factor alpha-1 (CBFA-1)/runt-related transcription factor 2 (RUNX2), osteocalcin, and alkaline phosphatase, following induction in osteogenic medium [19]. These results were paralleled by a 30-fold increase in matrix calcification suggesting the applicability of adipose tissue-derived stromal cells (ADSCs) to bone repair. Multiple studies into the BTE potential of ADSCs were published in the following years both* in vitro* [16, 53, 54] and* in vivo* in animal models [20, 55–58] and in humans [7, 8, 59]. Many studies noted not only the greater accessibility of ADSCs, but also the greater number of progenitors in lipoaspirates (100 times the number of progenitors compared to the same volume of BM) [60]. Animal studies not only revealed the potential of ADSCs to generate functional bone [16, 20, 56, 61] but also demonstrated additional advantages over bone marrow derived counterparts, such as a propensity for greater proliferation [62] and CFU-f formation [16, 58], reduced senescence* in vitro* [16, 54], and greater production of CXCL 12 [57], the so-called HSC-niche factor [63], and lower risk of malignant transformation [11].

The clinical application of ADSCs for BTE is followed rapidly with a case report of maxillary reconstruction. GMP-expanded ADSCs were induced with BMP-2, seeded onto a beta-tricalcium phosphate (*β*-TCP) scaffold, and implanted within the patient's rectus abdominis muscle. Eight months later, the scaffold and surrounding titanium cage were transferred to the patient's jaw. Vascularised living bone tissue was reported and dental implants were successfully made four months later [7]. In situations where little autologous bone is available, as in children, adipose tissue represents a good potential source of cells. Lendeckel and colleagues [59] reported the use of ADSCs to supplement autologous bone material in the successful repair of calvarial defects in a 7-year-old patient: bone grafts were mixed with fibrin glue and ADSCs were injected into the grafts in a single operational procedure. Applying similar techniques, 13 patients were treated with cultured ADSCs implanted on either bioactive glass (BAG), *β*-TCP, or “ChronOS” (Mathys, Switzerland) synthetic *β*-TCP granules, with or without the addition of BMP-2. In 10 of the 13 cases successful bone integration and repair was reported [8].

The unfractioned lipoaspirate, or stromal vascular fraction (SVF), “consists of a heterogeneous population of cells that includes not only adipose, stromal, and hematopoietic stem and progenitor cells, but also endothelial cells, erythrocytes, fibroblasts, lymphocytes, monocyte/macrophages and pericytes, among others” [64]. Using the SVF, an autogenic osteogenic graft prepared using a perfusion bioreactor system could be ready for implantation in 5 days, as compared to 3 weeks when using bone marrow derived cells [65]. Additionally, the greater proliferative capacity of SVF cells [58, 62] and the presence of vasculature-forming endothelial cells [65, 66] may permit their application to intraoperative procedures [17, 67], reducing operative duration and associated morbidity. Animal studies suggest that SVF holds merit as a viable BTE cell source [67, 68].

### 2.4. Definition of a Multipotent Mesenchymal Stromal Cell Population

Owing to doubts about the validity of comparisons made between different studies using stromal cells from different tissues, the International Society for Cellular Therapy (ISCT) outlined a set of minimal criteria for the identification of multipotent mesenchymal stromal cells, stipulating that the cells must be plastic-adherent, express CD105 (endoglin), CD73 (ecto-5′-nucleotidase), CD90 (Thy-1) and lack expression of CD45 (lymphocyte common antigen), CD34 (CD34^+^ cells were included in updated version of the statement to include SVF cells [64]), CD14, or CD11b (ITGAM), CD79a (MB1), or CD19 and HLA-DR surface molecules, and, finally, differentiate to osteoblasts, adipocytes, and chondroblasts* in vitro* [69].

A number of problems exist with these criteria: the use of plastic adherence as a requirement encourages the use of two-dimensional (2D) culture which has been associated with a loss of cell motility, proliferative activity [70], and osteogenic potential [71, 72]. The 2D environment alters cellular behaviour and may negatively affect both ADSC and BMSC development [73]. Furthermore, not all osteoprogenitors are necessarily adherent to culture dishes, BM-derived mesenpheres, for example [44]. The stipulation that* in vitro* cultured cells can be forced to differentiate into chondrocytes, osteocytes, and marrow adipocytes, following prolonged, constant concentrations of differentiation factors, is at odds with the variation over time in the levels of these agents* in vivo* (reviewed in [74]) and results suggesting that resident stem cell populations have an intrinsic tendency to differentiate into the lineages of their resident tissue [58, 75–77], perhaps through epigenetic programming [75]. This last point is exemplified by results indicating that skeletal genes are upregulated in undifferentiated BMSCs that are unchanged in ADSCs [78] and the same BMSCs require no induction to form bone/bone marrow* in vivo* [78], while other sources of stromal cells require chemical [18, 19, 79] or genetic [17] induction. This may be significant when one considers the potential effects of long-term exposure of cells to inductive agents; BMP-2, for example, has been linked to the malignant transformation of breast cells [12], ectopic bone growth [10], and neurotoxicity [13]. Consideration must be given also to the methods by which differentiation into the three skeletal lineages is assessed; initial studies which reported the successful differentiation of non-BM cells into skeletal lineages did so on the basis of one histological stain per lineage. The combined use of histology, surface markers, multiple gene analysis, or proteomics would make for a more robust analysis of cellular developmental state (as used by Murata et al. [20]). Lastly, while many studies have found the ISCT marker profile between ADSCs and BMSCs to be identical [78, 80], others have noted significant differences in the two cell populations particularly in the expression of CD106 [16, 64] and CD36 [64]. It seems clear that ADSC and BMSC are far from identical: a salient point is their differing propensity to form cartilage, bone, and fat tissues, possibly due to epigenetic factors [75]. Therefore the interchangeable use of “MSC” to describe both (as well as stromal cells derived from other tissues) is inaccurate, and its discontinuation has been called for [81, 82]. An indication of the cell source is crucial; thus “BMSC” and “ADSC” or term or a similar term ought to be used to clarify the tissue of origin at the very least.

### 2.5. BM and AT: Promising Cell Sources for BTE, but Not without Hurdles to Clinical Use

The demonstrated benefit of BMSC-based BTE [6, 51, 52] is backed up by a number of recent studies proposing candidates for the skeletal progenitor [43–45, 83] and others showing the innate osteochondral propensity of BMSCs [53, 78, 84, 85] (Table 1). The ability of the SSC within the BMSC population to generate a functional bone/bone marrow organ [4, 43, 84] places them as the prime candidate for regeneration of bone tissues. If the native physiological state of bone tissue is to be recreated then the ability to form the HME, where the SSC and HSC reside, is of paramount importance.

The clinical success of ADSC-based methods [7, 8, 20, 56] (Table 1) suggests that nonbone tissues can indeed be coaxed into forming mature bone. The lack of evidence for HME-support [86] casts doubt on the use of cells from this source, but given the evidence that they can be used to achieve successful bone repair coupled with the ease of collection and abundance (cf. BMSCs) [60, 72] the speed at which they can be prepared and replaced into the defect site [87] and their resistance to senescence [54, 88] and malignant transformation [89] ADSCs hold great potential for BTE.

Challenges facing the BTE field include the elucidation of the mechanisms underlying the developmental pathways involved in bone regeneration/repair and substantial task of bringing BTE technologies to the clinic at a cost that is on a par with current techniques.

## 3. Developmental Engineering

Initial hopes for the application of tissue engineering to the repair and regeneration of bone have not yet come to fruition. Unfortunately, the unmet clinical need which generated the enthusiasm surrounding TE in the 1990s [90] persists today [60]. Developments, particularly in animal models (see previous section), have advanced the field, but the resulting clinical impact has been limited.

### 3.1. Advancing the “Cells + Cytokines” Model of BTE

Traditionally, BTE has focused on tissue replacement through the* in vitro/ex vivo* generation of implants which effectively mimic the mature tissue as it is found in the adult. This has been achieved through the use of different cells, scaffold materials, and soluble factors to create a mechanical/biochemical profile that is similar to the tissue it is designed to replace [90]. Scaffolds give physical strength, durability, malleability, and three-dimensional structure, allowing for custom-sized implants with specific mechanophysical characteristics. The choice of scaffold is not insignificant as the architecture, rigidity, and biochemical properties can modulate cell differentiation. Different scaffold materials can be combined [91] or supplemented with growth factors such as BMPs [10]. Various combinations of growth factors are routinely used to guide cell differentiation towards the desired phenotype; however the use of a limited number of factors is a long way from the complexity seen* in vivo* [60, 92]. Bioreactors using controlled perfusion of media through three-dimensional scaffolds recapitulate, to some degree, mechanical [93, 94] and hydrostatic forces [95], representing a step towards replicating the tempospatial complexity of the* in vivo* microenvironment, something which may well be impossible to recreate* in vitro*.

In recent years a number of laboratories have adopted strategies which do not conform to the standard “cells + scaffold + cytokines” approach that typifies the majority of BTE studies, instead opting for a “developmental engineering” (DE) approach [21, 22]. Just as the transition from two-dimensional to three-dimensional* in vitro* cell culture [72] recognised the merits of more faithfully replicating* in vivo* spatial relationships [70], the transition from TE to DE attempts to take into account the complexity of* in vivo* developmental processes and to incorporate features found therein for the design and generation of developmental templates.

### 3.2. Key Concepts of Developmental Engineering

Recently, Lenas et al. [21, 22] described a fusion of engineering principles and concepts from developmental biology, which they termed “developmental engineering.” The authors outlined the utility of applying concepts such as path-dependence, robustness, and modularity, to the manufacture of tissue grafts/implants. Robustness, within the context of developmental processes, refers to the ability of a system to function consistently despite external fluctuations. A robust developmental mechanism would therefore be able to cope with a degree of dissimilarity between the native tissue and the implant. A problem encountered when trying to gauge the characteristics necessary for successful stimulation of native repair processes is one of sensitivity; the basic tools and the limited sensitivity of currently applied methods means we are not yet able to predict whether a certain implant will function effectively, leading to much trial and error. However, the modularity of many developmental processes permits* ex vivo* experimentation to determine optimal conditions and timing for implantation to achieve the best results* in vivo* [84, 96]. Additionally,* ex vivo* experiments can be used to identify markers for the successful completion of multistage developmental processes [22, 97]. In this fashion, the progress of the implant can be monitored,* in vivo*, through the stages of development, highlighting where problems lie and thus where refinement is needed. The successful completion of each step of development sets the stage for the next step, providing optimal conditions. This concept, rooted in economics, law [98], and biology, is called path-dependence and describes a situation where the outcome of one process directly influences the effectiveness of a successive process. Thus, one process acts as a check-point for the correct completion of the previous step, and at the same time completion of the previous step sets the stage for the following stages. In the context of bone regeneration, this is exemplified by hypertrophic chondrocytes which act as a natural scaffold for osteogenesis as well as secreting factors which orchestrate the differentiation of osteoblasts from perichondrial cells, as well as the mineralisation and vascularisation of the neo-bone tissue, restoring normoxic conditions required for optimal bone growth and bringing vital materials [99]. This concept has experimental support; hypertrophic chondrocytes have been shown to stimulate bone regeneration* in vivo*, while lesser developed tissues were not as effective in stimulating the formation of bone tissue, likely reflecting the path-dependence of this process [28, 84]. Indeed, BMSCs have been demonstrated to follow the endochondral route when chondrogenically primed and implanted in a vascularised tissue [25]. The use of a hypertrophic differentiation medium accelerates and makes the process more efficient.

Instead of aiming to phenocopy the adult tissue-state, researchers are drawing on the work of developmental biology, which states that “normal tissue healing in the adult involves progressive remodelling of pre-existing tissue structures” [90] to generate grafts that recapitulate the immature tissue-state. By implanting the precursor state of a tissue, or “organ germ” [57, 100], elements of the implant can interact with natural developmental cues to regulate differentiation and growth and to provide cues for cell invasion, remodelling, and revascularisation in the correct spatiotemporal context. In this manner we might overcome one of the greatest challenges facing TE, that is, effectively mimicking the complexity of natural developmental processes, thereby leading to formation of an authentic mature tissue.

Considering that the vast majority of bones develop through endochondral ossification, an endochondral approach to bone regeneration is now considered “developmental engineering.” However, the endochondral approach* per se* does not make “developmental engineering” a bone regeneration strategy. In fact, flat bones of the head develop through intramembranous ossification.

### 3.3. Recapitulation of Endochondral Ossification through Chondrogenic Differentiation

Historically, TE has directed the formation of neo-bone through the intramembranous route relying on the presence of mineralised substrate scaffolds to initiate bone growth through intramembranous ossification; however more recently numerous studies have illustrated the advantages of bone formation through endochondral ossification [25, 29, 41, 84, 91, 96, 101, 102]. Endochondral ossification is the method by which the axial and long bones of the skeleton (the vast majority of bones) are formed during embryogenesis [103] and has many features common to bone regeneration after fracture [104, 105] including activation of key signalling pathways such as Indian hedgehog (IHH), parathyroid-related hormone protein (PTHrP), wingless (wnt), and BMPs (although, notably, the postnatal environment differs from that of the developing embryo [104]). The process entails the condensation (clustering together through cell surface receptors and adhesion molecules [106]) of chondrocytes, which secrete a collagenous (type II) matrix rich in proteoglycans. Under the control of two of the master regulators of bone development, IHH, and PTHrP (see [103]), chondrocytes at the centre of the proto-bone organ cease to proliferate and become enlarged (hypertrophic), producing large amounts of type X collagen, directing initial mineralisation [107] and vascularisation through VEGF production, before undergoing apoptosis, to leave a cartilage scaffold that will eventually be remodelled into mature bone [103]. This strategy has been exploited for bone regeneration; implantation of hypertrophic huBMSCs in nude mice has been demonstrated to lead to the growth of ectopic bone structures as a result of human cells playing an active role of osteogenesis [25]. BMSCs embedded in *β*-TCP scaffolds were able to generate frank bone* in vivo*, but chondrogenic priming was necessary for the production of bone + BM [96], while huBMSCs seeded on collagen type I scaffolds induced towards endochondral ossification formed not only bone organs, but also a fully functional BM which was shown to sustain haematopoiesis in lethally irradiated mice [84]. In a previous study cells that were not hypertrophic at the time of implantation failed to generate bone and were resorbed, indicating that the developmental stage is a critical factor in dictating whether the implant will proceed to the next stage [25, 108].

There are multiple advantages to implanting chondrogenically primed cells: chondrocytes are more likely to survive the hypoxic* in vivo* environment [101]; they stimulate vascularisation [101, 109] through secretion of VEGF [109] and have been shown to increase bone formation* in vivo* through BMP production [60]. Additionally, by selecting a stating material which most closely matches the* in vivo* precursor to the tissue of interest and by guiding those cells through developmental stages using known markers, an intermediate form of the tissue is generated which “contains all the necessary and sufficient instructive elements for its regeneration” [110]. This has obvious implications for the choice of cell source, since a cell containing detectable genetic, epigenetic, proteomic modifications which are optimal for a particular developmental path is not only more likely to produce a higher quality final product, but also likely to contain additional characteristics which the limited and basic range of tests at our disposal cannot gauge. That said, ADSCs, which had low intrinsic bone-forming potential and produced no neo-bone in their uninduced state, when chondrogenically primed deposited a proteoglycan-rich cartilaginous matrix and were able to generate a similar amount of bone as uninduced BMSCs [62]. This suggests that, by rerouting ADSCs through endochondral ossification, a precursor state is created that favours bone formation. It is noteworthy that despite the successful rerouting of ADSCs, uninduced BMSCs achieved better final results, perhaps reflecting intrinsic factors that predispose them to bone formation [62, 75].

### 3.4. Considerations and Limitations of Mimicking Embryogenesis for BTE

While the adoption of processes which mimic embryogenesis has demonstrated merit [84, 96], there are salient physical, biochemical, mechanical, and immunological differences between the developing embryo and a mature tissue microenvironment [60, 92, 104, 111]. Accordingly, we must adjust the design of prospective implants to reflect these differences [26]. Embryonic development occurs under different immunological and inflammatory settings as well as at a much smaller scale than in the adult; both of these factors must be addressed if embryonic processes are to be harnessed for the successful engineering of bone grafts.

Paracrine signalling gradients which function at the embryonic scale are likely to be inefficient in a much larger graft. Modular implants, comprising many smaller units, may be utilised to overcome this hurdle (modular implants-cellular sheets [112]) in addition to addressing some of the limitations of mass transfer such as necrosis at the core of the engineered tissue.

The immunological milieu controlling developmental processes and the influx of cells at the embryonic stage of bone growth remains to be fully elucidated. This is likely to be a crucial step if we are to fully harness the potential of developmental engineering, as immune factors are significant mediators of bone healing and regrowth [104], which can result in retardation of healing if suppressed [111, 113]. Interestingly, this last point serves to highlight the differences between developmental processes underway during embryogenesis and those involved in the adult: while inflammation represents one of the main drivers of bone repair [84, 111], it is absent during normal bone development. In fact, the significance of interleukin-1*β* (IL-1*β*) in the revascularisation, mineralisation, and cartilage remodelling activity of huBMSCs has been illustrated [111, 114].

In conclusion, the adoption of a developmental engineering paradigm for the regeneration of bone represents a potential method to mitigate the enormous hurdle presented by largely unknown* in vivo* complexity. By generating precursor organ germs based on observable* in vitro* elucidated markers and allowing natural cues to orchestrate the development of hypertrophic chondrocyte templates, it is foreseeable that future bone repair strategies will achieve clinical use. However, if we are to effectively utilise this technique, a clearer more complete understanding of the biochemical and mechanical forces at work in both the developing embryo and the adult is required.

## 4. Implications of Scaffold Technology for Cell versus Cell-Free Approaches

With the objective of repairing bone in a manner which recalls natural healing processes, both cell-based and cell-free methods have been utilised: both have advantages, but currently cell-based therapeutic strategies are the* status quo.* This usually comprises BMSCs which have been extracted and either reinjected intraoperatively or cultured* ex vivo* for several passages to generate many more cells which are then reinjected in their current state, or, more commonly, seeded on a three-dimensional scaffold material. Innovations in the preparation of scaffold materials have added an additional dimension to current BTE treatments and may pave the way to standardized, off-the-shelf* in vitro* derived cell-free products in clinical bone repair.

### 4.1. Cells or No Cells?

Cell-based strategies, most often utilising BMSCs, have been shown to be more successful at stimulating bone healing than cell-free approaches, resulting in greater mineralisation, ossification, and increased angiogenic potential [27–29, 48, 49]. These results are supported by data showing cell-based techniques to be clinically advantageous [115, 116]. However, the downsides to autologous cell-based therapy are significant and can be prohibitive in some cases. The rarity of BMSCs can be limiting to the point of rendering cell extraction unfeasible (especially in the elderly and the ill) and too few CFU-f within a BM extract will fail to generate neo-bone tissue [72, 117]. Even in healthy individuals, cell extraction requires an additional procedure which carries added morbidity. Eliminating the need for extra surgery has strongly motivated the development of intraoperative techniques which, while avoiding the time-expensive and laborious GMP handling of cells in the laboratory, are also limited by the number of BMSCs available for reinjection.

Cell-free technologies have been proposed as an alternative to sidestep many of the barriers associated with cell-based techniques for bone-specific and other areas of tissue engineering. A product which is available “off-the-shelf” following decellularisation and sterilisation has obvious practical advantages from a surgical perspective such as the reduction of intrapatient variability and would allow the selection and preparation of the implant prior to surgery. Additionally, the implant can be recellularised with autologous BMSCs prior to use if sufficient cells are available [29]. Also, a sterile acellular product would be amenable to storage and thus easily transported to areas of need where the resources for preserving cell-based products might be lacking. All of these reasons would act to increase the clinical uptake.

### 4.2. Decellularised ECM as a Biological Scaffold for BTE

Many recent studies have attempted to mimic the inherent complexity of the biological microenvironment, in terms of architecture and biochemical constituents, through the use of decellularised extracellular matrix (ECM) from a variety of animal sources, both human [118, 119] and nonhuman [120, 121]. The latter option presents the possibility of benefiting from existing slaughter processes to access a large volume of material for decellularisation. The risk of zoonoses, especially prion diseases, can be reduced by sourcing animals from prion-free island populations [122, 123]. The use of cell lines derived from either human or nonhuman animals to produce a functional ECM that could subsequently be decellularised presents the possibility of standardisation, reducing donor-to-donor variability [9].

For the successful application of allogenic or xenogenic sources, the implants must be effectively decellularised to avoid a damaging immune response. The decellularisation protocol represents a balancing act between preserving the native biochemistry and microstructure and simultaneously removing cells and other immunogenic materials. Decellularisation is achieved primarily through physical/mechanical (predominantly freeze-thaw), chemical (including detergent-based methods), and enzymatic means coupled to wash steps to remove debris (extensively reviewed in [124]). Any treatment is almost certain to disrupt the native structure, either physically or biochemically, and therefore strip away many of the growth factors, cytokines, and inflammatory factors harboured within the ECM.

With regard to bone engineering, the modern concept of developmental engineering suggests that the endochondral route provides the optimal template. Previously, hypertrophic chondrocytes derived from human BMSCs were shown to be remodelled and replaced by frank bone tissue, including a functional haematopoietic compartment [24]. Accordingly, decellularised hypertrophic cartilage has been used as a template to stimulate the regeneration of bone material through endochondral pathways, promoting the invasion and proliferation of host cells [27–29]. Induced apoptosis of hypertrophic chondrocytes has recently been proposed to decellularise ECM for bone regeneration through the retroviral transduction of a chemically inducible caspase-9-bearing construct. This was shown to be superior to a standard freeze-thaw protocol for the regeneration of bone. Postapoptotic cartilage and implants containing live BMSCs, but not nonhypertrophic cartilage, underwent extensive remodelling and after 12 weeks* in vivo* tested positive for the presence of a BM space, although implants containing live cells outperformed the “apoptosed” tissue [27]. Elsewhere, nonhypertrophic cartilage was shown to be inferior to hypertrophic constructs in a mouse femoral defect model, where only the latter were successful in bridging the defect [28].

To date, the use of cell-free techniques has yet to demonstrate equivalence to cell containing preparations. Developments in the methods used for decellularisation will undoubtedly result in more effective scaffold materials, due to greater retention of ECM-associated molecules with simultaneous removal of cellular material, to yield bone engineering products with off-the-shelf convenience, as well as low-maintenance storage, and increased customisation. Advances in scaffold preparation techniques, with or without autologous cells, likely represent an area of keen future research interest.

## 5. Conclusions

Intrinsic bone repair mechanisms are highly effective, but in certain cases external intervention is required. In some instances, BTE has been shown to provide clinical relief, but improvement in BTE technologies is required to allow its application to greater numbers of patients, particularly those to whom traditional bone grafting procedures are unfeasible.

Recent strategies in bone repair and regeneration have sought to embrace a developmental engineering approach, following as closely as possible the natural processes of bone development through the remodelling of hypertrophic cartilage templates* via* endochondral ossification. The predominant use of BMSCs for bone formation following the endochondral route reflects the role of the BM as the natural reservoir of skeletal-tissue forming cells, namely, the SSC, and illustrates their propensity to differentiate into skeletal lineages.

BMSCs form bone + BM* in vivo* which is essential if creation of the HME is required. The factors (genetic, epigenetic, proteomic, etc.) responsible for the predestination of BMSC to form functional bone + BM are unknown, and we cannot currently quantify the extent of these “unknown unknowns” [125]. Likely a fraction of these factors driving BMSC to form bone + BM will also regulate additional steps in skeletal development and remodelling allowing cells to correctly react to autocrine and paracrine developmental signals. Until we have a clearer understanding of the mechanisms underlying bone development, BMSCs represent a more rational choice for bone regeneration and repair if long-term propagation of bone tissues (and haematopoietic cells) is desired.

This last point assumes the availability of autologous BMSCs, which is not always the case. ADSCs are championed by their proponents for their far greater accessibility and the presence of greater numbers of CFU-f per unit volume than that found in the BM. Clinical evidence of the efficacy of ADSC-based therapy indicates that AT is an excellent source for cells for the generation of bone tissue. Is this a question of quantity over quality though? Where simple bone tissue is called for rather than a functional bone-BM organ, it may be the case that ADSC-derived bone is “good enough.” This, coupled with the great advantages of using ADSCs, may be enough to ensure the continued application and development of ADSCs to bone repair.

Regardless of cell source, currently live cell-based implants tend to be superior to cell-free and decellularised alternatives at regenerating bone tissue. Recent advances in decellularisation protocols are bringing the performance of decellularised and devitalised tissues to ever greater levels, approaching that of vital implants, with the added value of storage, transportation, and the possibility of allogenic or xenogenic-derived grafts to circumgate the difficulties in obtaining autologous cells for bone regeneration and repair. Another valuable strategy to improve the results of decellularised matrices is based on the intraoperative recellularisation of the graft. In fact, it is nowadays possible to recellularise a decellularised matrix with autologous bone marrow cells, or fat-derived stromal and vascular cells. Future research should be focused on developing effective and sustainable clinically compliant bone regeneration strategies that combine the efficacy of cell-based therapies with the superior practical features of decellularised matrices.

## Figures and Tables

**Table 1:** Highlights of selected publications regarding the osteogenic potential of various cell sources.

Author, year	Model	Cell source	Treatment	Scaffold	Outcome	Criteria
Muraglia et al., 2000 [41]	*In vitro*	Human BMSC clones	1 week complete medium (DMEM, 10% FBS: CM) ± FGF-2 followed by 1–3 weeks in chondrogenic, adipogenic, or osteogenic medium	NA	17% (FGF−) and 34% (FGF+) displayed the potential to originate all three phenotypes induced 80% (FGF−) and 60% (FGF+) were able to undergo osteogenesis and chondrogenesis 3% (FGF−) and 5% (FGF+) underwent osteogenesis only No clones underwent solely chondro- or adipogenesis	Osteogenesis: anti-osteocalcin IHC Chondrogenesis: anti-CNII IHC Adipogenesis: Sudan Black staining

Zuk et al., 2001 [18]	*In vitro*	Human PLA	PLA cells at passage 1 were differentiated for 2–6 weeks in chondrogenic, adipogenic, osteogenic, or myogenic medium	NA	Chondrogenesis: positive staining for Alcian Blue and coll-II in chondrogenically differentiated cells Adipogenesis: 42% cells were Oil red O positive. Osteogenesis: 50% PLA cells positive for AP staining and Von Kossa staining (calcification) present in osteogenically differentiated cells, but not undifferentiated PLA cells Myogenesis: myogenic differentiated cells expressed MyoD1 and myosin	Chondrogenesis: Alcian Blue stain and collagen II-specific mAB Adipogenesis: Oil red O stain Osteogenesis: AP and Von Kossa staining Myogenesis: phase contrast microscopy, myosin- and MyoD1-specific mAB

Quarto et al., 2001 [6]	Human (*n* = 3)	Autologous human BM	Autologous BMSCs cultured *ex vivo* in CM + FGF-2	HA	All patients recovered limb function. After 27, 16, and 15 months, the patients reported no problems with the implants. Callus formation at implant site and integration with surrounding bone	Functional use of limbs. CT and radiograph to assess bone density and callus formation

Zuk et al., 2002 [19]	*In vitro*	Human PLA	PLA cells at passage 1 were differentiated for 9 hours (neurogenic) or 2–6 weeks in chondrogenic, adipogenic, osteogenic, myogenic, or neurogenic medium	NA	Chondrogenesis: confirmed by positive AB staining, positive IHC (KS, CS, CNIIb, and CN10) Adipogenesis: confirmed by increased GPDH, LPL, aP2 leptin, and GLUT4 activity, plus Oil red O staining Osteogenesis: confirmed by increased matrix mineralisation and osteogenic markers versus noninduced BMSCs Myogenesis: confirmed through increased expression of myogenic markers versus noninduced controls Neurogenesis: uncertain. Positive detection of nestin, NSE, and NeuN. Negative for neuronal markers: GalC, GFAP, MAP-2, NF-70, ChaT, GAD65, and MBP	Chondrogenesis: IHC against KS, CNII, and CS. WB for CNII, AG, and CN10 Adipogenesis: increased Oil red O stain, GPDH, leptin, GLUT4, PPARy2, and LPL expression Osteogenesis: matrix mineralisation, AP enzyme activity, RT-PCR (OC, CBFA-1, AP, ON, OP, BMP-2, c-fos, CNI, PTHR, RXR-a, and VDR), and WB (OP, ON, CNI, AP, RARa, and VDR) Myogenesis: RT-PCR (myod1, myf6, myf5, myosin), WB (DES, myod1, MG, MYF, and myosin heavy chain) Neurogenesis: IHC (NSE, NeuN, GFAP, and GalC), RT-PCR (nestin, ChaT, GAD65, GFAP, and MBP)

Hicok et al., 2004 [55]	Immunodeficient SCID mice	Human PLA	PLA was washed and maintained in CM followed by 3, 7, or 14 days in CM or osteogenic differentiation medium (OM)	HA-TCP	*In vitro*: increased AP activity in osteodifferentiated cells. Positive Alizarin Red staining in osteodifferentiated cells versus controls *In vivo*: more osteoid formation in implants + PLA than implants+ noninduced BMSCs	AP activity and Alizarin Red staining (matrix mineralisation) before implantation. *In vivo*: H&E staining

Warnke et al., 2004 [51]	Human (*n* = 1)	Autologous BM	BM + BMP7 + bone mineral blocks encased in a metal cage implanted ectopically for 7 weeks	Bovine bone mineral blocks	Vital neo-bone detected at 4 weeks, after implantation. 11 days after transplantation, bone remodelling and mineralisation were detected. Jaw function (mastication) was restored by the procedure	Bone growth detected by skeletal scintigraphy following injection of radioactive tracer

Huang et al., 2005 [85]	*In vitro*	Matched human (*n* = 5) AT and BM	Chondrogenesis induced by aggregate culture	NA	More cartilage-specific ECM deposited by BM cells than AT. Cells with appearance of hypertrophic chondrocytes seen in BM but not AT deposits More than twofold greater GAG levels in BM versus AT. 500–5000x higher CN10 levels in BM versus AT deposits	Chondrogenesis: GAGs assessed by toluidine blue stain and DMMB assay, and IHC (CNII, CN10) Adipogenesis: Oil Red O staining Osteogenesis: Von Kossa staining for calcified ECM

Im et al., 2005 [53]	*In vitro*	Nonmatched human AT (*n* = 6) and BM (*n* = 8)	Osteogenesis induced using OM (2-3 weeks) Chondrogenesis induced through pellet/fibrin culture	NA (2D culture)	Greater AP and Von Kossa staining in BMSCs versus ADSCs. BMSCs produced more proteoglycan and CNII	Differentiation was assessed using a semiquantitative histological grading system

Kern et al., 2006 [16]	*In vitro*	Nonmatched human AT (*n* = 18), BM (*n* = 18), and UCB (*n* = 59)	Cells were cultured in OM (2.5 weeks) or adipogenic differentiation medium (AM) Chondrogenesis induced through pellet/fibrin culture	NA (2D culture)	71% BM, 79% AT, and 100% UCB samples positive for osteogenesis 100% BM, 94% AT, and 0% UCB positive for adipogenesis 100% samples positive for chondrogenesis	Osteogenesis: AP and Von Kossa stains Adipogenesis: Oil red O Chondrogenesis: Safranin O Surface markers (CD43, CD73, CD90, CD14, CD34, CD45, CD105, CD133, CD29, HLA I, HLA II, CD106, and CD44) were also used

Sacchetti et al., 2007 [43]	Immunodeficient nih/nu/xid/bg mice	Human BM	Cultures were grown in aMEM + 20% FBS prior to implantation for 4, 7, and 8 weeks	HA-TCP + fibrin gel	BMSCs but not muscle and skin fibroblasts formed bone + BM. Human trabecular bone and periosteal cells formed bone but no BM *in vivo* BMSC CFU-f cells are uniquely CD146+ and can regenerate CD146+ CFU-fs *in vivo*	Bone and BM formation: H&E staining CD146 (and other surface markers) assayed by FACS and tissue immunostaining

Mesimäki et al., 2009 [7]	Human (*n* = 1)	Autologous AT	Cells expanded *ex vivo*, mixed with *β*-TCP in DMEM, 15% autologous serum + BMP-2 implanted ectopically for 8 months	*β*-TCP	After 8 months *in vivo*, the implant was vascularised and resembled mature bone	Bone was analysed radiologically

Evans, 2015 [17]	Rabbit femoral condylar and trochlear groove defect	Rat	Muscle and fat transduced with human BMP-2	NA	Transduced muscle implants significantly improved healing after 6 weeks	RT-PCR: OP, CNIaI, BSP, OC, AP, and CBFA1 Histology: AB and Safranin O CT: bone remodelling

Vishnubalaji et al., 2012 [58]	*In vitro*	Nonmatched human AT (*n* = 5) and BM (*n* = 5)	Cells were induced towards osteogenic and adipogenic fates	NA (2D culture)	Osteogenesis induced in both cell groups. Greater AP activity, mineralisation, and significantly higher levels of OC and OP in BM versus AT cells	Osteogenesis: AP, Alizarin Red S, Von Kossa stains. Calcium levels assayed Adipogenesis: Oil red O stain RT-PCR: aP2, PPARy, AP, AN, OP, and OC Surface markers: CD13, CD31, CD73, CD105, CD44, CD29, CD90, CD146, CD34, CD45, CD14, and HLADR

Brocher et al., 2013 [62]	Immunodeficient SCID mice	Nonmatched human AT (*n* = 7) and BM (*n* = 7)	*In vitro* expansion ± chondrogenic preinduction	*β*-TCP or no scaffold	All preinduced BM-samples generated neo-bone after 8 weeks *in vivo* ± *β*-TCP. AT samples were dependent on *β*-TCP for bone formation in preinduced samples producing bone in 13/18 samples	Histology: TB, Safranin O, H&E, Movat's pentachrome, and Masson's trichrome

Sándor et al., 2014 [8]	Human (*n* = 13) craniomaxillofacial defects	Autologous AT	Expanded *in vitro* and embedded in a scaffold ± BMP-2	Bioactive glass or *β*-TCP	Successful integration with surrounding bone noted in 10/13 cases. 1 failed case due to patient nose picking	Postoperative CT and radiographs at 12–52 months follow-up

Murata et al., 2015 [20]	Porcine femoral trochlear defect (*n* = 2)	Autologous AT	*In vitro* culture of cell spheroids moulded into cylinders	NA	Results varied with regard to cartilage, but implant sites showed better remodelling of subchondral bone than control sites	Monthly CT scans Macroscopic IRCS grading, IHC, and histology of implants at 6 and 12 months

Reinisch et al., 2015 [78]	Immunodeficient NSD mice	Nonmatched human AT (*n* = 5), BM (*n* = 5), UCB, and skin-derived cells	Cells were expended and loaded onto scaffolds. After implantation, mice were given PTH daily	HA-TCP powder	All cells had similar phenotypes *in vitro*, but only BM-derived cells formed bone + BM *in vivo*BM-derived cells had a distinct gene expression and methylation signature suggesting skeletal proclivity	Histology, H&E, and pentachrome stains Gene expression and methylation performed using microchip arrays

## Abbreviations

*β*-TCP: Beta tricalcium phosphate
HA: Hydroxyapatite
PLA: Processed lipoaspirate
PTHrP: Parathyroid hormone related protein
SVF: Stromal vascular fraction
AB: Alcian Blue
ADSC: Adipose-derived stromal cell
AG: Aggrecan
AN: Adiponectin
AP: Alkaline phosphatase
AT: Adipose tissue
BM: Bone marrow
BMSC: Bone marrow stromal cell
BSP: Bone sialoprotein
BTE: Bone tissue engineering
CBFA-1: Core-binding factor alpha 1
ChaT: Choline acetyltransferase
CNI: Collagen type I
CNII: Collagen type II
CN10: Collagen type 10
CS: Chondroitin-4-sulfate
DE: Developmental engineering
DES: Desmin
ECM: Extracellular matrix
GLUT4: Glucose transporter type 4
GPDH: Glycerol-3-phosphate dehydrogenase
HME: Haematopoietic microenvironment
HSC: Haematopoietic stem cell
ICRS: International Cartilage Repair Society
IHC: Immunohistochemistry
KS: Keratan sulfate
LPL: Lipoprotein lipase
mAB: Monoclonal antibody
MBP: Myolin basic protein
MG: Myogenin
MYF: Myogenic regulatory factor
NeuN: Neuronal nuclei protein
NSE: Neuron-specific enolase
OC: Osteocalcin
ON: Osteonectin
OP: Osteopontin
PPARy: Peroxisome-proliferating associated receptor-y
PTH: Parathyroid hormone
PTHR: Parathyroid hormone receptor
RARa: Retinoic acid receptor alpha
RT-PCR: Real time PCR
RXRa: Retinoid X receptor alpha
SSC: Skeletal stem cell
TE: Tissue engineering
UCB: Umbilical cord blood
VD: 1,25-Dihydroxyvitamin D3
VDR: Vitamin D receptor
WB: western blotting.
